# *MAML2*‐Rearranged Hidradenoma of the Breast: Clinicopathologic and Molecular Analysis of Four Patients

**DOI:** 10.1155/crip/3861274

**Published:** 2026-01-30

**Authors:** Naomi Gondo, Yasuyo Ohi, Tsubasa Hiraki, Akira I. Hida, Shuichi Kanemitsu, Yasuaki Sagara, Shinji Ohno

**Affiliations:** ^1^ Department of Breast and Thyroid Surgical Oncology, Sagara Hospital, Kagoshima, Japan; ^2^ Department of Pathology, Sagara Hospital, Kagoshima, Japan; ^3^ Department of Diagnostic Pathology, Shizuoka Cancer Center Hospital, Nagaizumi, Japan, scchr.jp; ^4^ Department of Pathology, Matsuyama Shimin Hospital, Matsuyama, Japan

**Keywords:** breast hidradenoma, case series, fluorescence in situ hybridization (FISH), *MAML2* gene rearrangement, sweat gland tumor

## Abstract

Hidradenoma (HA) is a rare, benign adnexal tumor typically arising in the skin, with only a few reported cases in the breast. Histologically, HA displays solid and cystic patterns with diverse cellular components. Although *MAML2* gene rearrangement is observed in some cutaneous HAs, its significance in breast HA remains unclear. We retrospectively analyzed four cases of breast HA at our institution, focusing on clinicopathologic and molecular characteristics. *MAML2* rearrangement was detected in all cases by FISH, suggesting it may be a defining feature of breast HA. Because breast HA frequently mimics malignancy and is often diagnosed only after excision, heightened awareness and recognition of its distinctive clinicopathologic and molecular features are essential to prevent overtreatment.

## 1. Introduction

Hidradenoma (HA) is a type of skin adnexal tumor characterized by differentiation toward sweat ducts and glands. Histologically, HA is characterized by an intradermal solid or solid–cystic proliferation composed of pale or clear cells, as well as other cell types, including polygonal, mucinous, squamoid, oxyphilic/oncocytic, and epidermoid cells. Reflecting its complex histological diversity, HA has been referred to by several different names, such as eccrine acrospiroma, nodular HA, clear cell HA, or solid cystic HA.

When HA occurs in the breast, it is extremely rare; only a few cases have been reported in the English literature [1–8]. This rarity poses a diagnostic challenge because its imaging and cytologic features often mimic malignancy and may result in overtreatment.

A growing body of evidence suggests that *MAML2* gene rearrangement plays a crucial role in the tumorigenesis of HAs and related glandular tumors. The translocation t(11;19)(q21;p13) involving the *MAML2* gene has been detected in some cases of mucoepidermoid carcinomas (MECs) of the salivary gland and bronchial glands, as well as in HAs of the skin [9, 10]. This leads to the *CRTC1–MAML2* fusion, which alters Notch and CREB signaling and promotes tumor development [9, 11–13].

However, the genetic profile of breast HA remains poorly understood, and the significance of *MAML2* gene rearrangement in this tumor type has yet to be fully elucidated. If *MAML2* gene rearrangement is consistently detected in breast HA, it may represent a characteristic genetic alteration that, in combination with other pathological features, contributes to its distinction from other benign and malignant breast tumors.

We present four cases of breast HA, all of which harbored *MAML2* rearrangement. Our findings support the notion that this genetic feature may be a molecular hallmark of breast HA, aiding accurate diagnosis and avoiding unnecessary treatment.

## 2. Case Description

Over a 21‐year period, a total of 15,609 breast lesions were resected in our hospital. Among 1840 benign lesions, four cases were identified as HA. The incidence of HA among benign lesions was therefore 0.2%. This frequency may be influenced by selection bias, as only resected benign lesions were included in the denominator, often due to diagnostic uncertainty or progressive growth.

We report four cases of HA of the breast in female patients aged 38–55 years, each presenting distinct clinical features but demonstrating shared histopathologic and molecular characteristics. Clinical and pathological findings are summarized in Tables 1 and 2.

**Table 1:** Clinical features, imaging findings, and pathological diagnoses of four patients with hidradenoma of the breast.

**Case**	**Age (year)**	**Sex**	**Presentation**	**Location**	**Size (mm)**	**Imaging**	**Biopsy**	**Diagnosis**	**Treatment**	**Follow-up (months)**
1	38	Female	Palpable lump, no discharge	Left breast, 2 o′clock position	20	Mammography: Well‐defined mass Ultrasound: Complex solid and cystic mass	Core needle biopsy	Clear cell sweat gland adenoma or low‐grade DCIS	Excision	86
2	38	Female	Palpable lump, no discharge	Left breast, 12 o′clock position	15	Mammography: No findings Ultrasound: Complex solid and cystic mass	Core needle biopsy	Clear cell hidradenoma, possibility of low‐grade DCIS	Excision	12
3	55	Female	Not palpable	Left breast, 12 o′clock position	8	Mammography: No findings Ultrasound: Hypoechoic mass and echo‐free area	Fine‐needle aspiration	Suspected malignant tumor	Excision	168
4	43	Female	Abnormal nipple discharge	Right breast, 3 o′clock position	10	Mammography: Partially spherical, indistinct mass Ultrasound: Intraductal tumor in the breast	Fine‐needle aspiration	Suspected malignant tumor	Excision	170

**Table 2:** Histopathological and immunohistochemical features of hidradenoma in the breast compared to previously reported cases.

**Case**	**Solitary/multiple**	**Solid and cystic appearance**	**In situ component**	**Clear cells**	**Eosinophilic cell**	**Cuboidal cells lining the ductal/glandular structure**	**Immunohistochemistry of clear or eosinophilic cells**				**Immunohistochemistry of cuboidal cells**		**Genetic study**
							**CK5/6**	**CK7**	**p63**	**SOX10**	**p63**	**SOX10**	
Hsieh et al. [7]	Solitary	+	Unknown	+	+	+	+	NP	+	−	−	+	*MAML2* break‐apart FISH
Memon et al. [8]	Solitary	+	Unknown	+	+	+	+	+	+	−	−	+	*MAML2* break‐apart FISH
Kazakov et al. [6]	Solitary	+	Unknown	+	+	−	NP	NP	NP	NP			RT‐PCR
Knoedler et al. [14]	Solitary	+	Unknown	+	+	−	NP	NP	+	NP	NP	NP	Karyotype analysis
Black et al. [12]	Solitary	+	Absent	+	+	+	+	+	+	−	−	+	*MAML2* break‐apart FISH+RNA‐based fusion testing
	Solitary	+	Absent	+	+	+	+	+	+	−	−	+	*MAML2* break‐apart FISH+RNA‐based fusion testing
	Solitary	+	Absent	+	+	+	+	+	+	−	−	+	*MAML2* break‐apart FISH+RNA‐based fusion testing
	Solitary	+	Conventional DCIS	+	+	+	+	+	+	−	−	−	*MAML2* break‐apart FISH+RNA‐based fusion testing
	Solitary	+	Absent	+	+	+	+	+	+	−	−	−	*MAML2* break‐apart FISH+RNA‐based fusion testing
	Solitary	+	Absent	+	+	+	+	+	+	−	−	−	*MAML2* break‐apart FISH+RNA‐based fusion testing
	Solitary	+	Absent	+	+	+	+	+	+	−	−	+	*MAML2* break‐apart FISH
	Solitary	+	Absent	+	+	+	+	+	+	−	−	−	*MAML2* break‐apart FISH
Luo and Hu [13]	Solitary	+	Absent	+	+	+	+	+	+	−	−	+	*MAML2* break‐apart FISH
Our cases													
Case 1	Solitary	+	Absent	+	+	+	+	+	+	−	−	+	*MAML2* break‐apart FISH
Case 2	Solitary	+	Absent	+	+	+	+	+	+	−	−	−	*MAML2* break‐apart FISH
Case 3	Solitary	+	Absent	+	+	+	+	NP	+	−	−	+	*MAML2* break‐apart FISH
Case 4	Multiple	+	Absent	+	+	+	+	NP	+	−	−	+	*MAML2* break‐apart FISH

### 2.1. Case 1

A 38‐year‐old woman presented with a palpable lump in the left breast (2 o′clock position), measuring 20 mm in diameter. Mammography revealed a well‐defined mass (Figure 1a), and ultrasonography demonstrated a complex solid and cystic lesion (Figure 1c). Core needle biopsy (CNB) suggested either a clear cell sweat gland adenoma or low‐grade ductal carcinoma in situ (DCIS). The patient underwent surgical excision. Histologically, the tumor showed both solid and cystic growth patterns composed of clear and eosinophilic cells, with duct‐like structures. Immunohistochemistry (IHC) was positive for p63, CK5/6, and CK7 and negative for ER. Fluorescence in situ hybridization (FISH) confirmed *MAML2* gene rearrangement. No recurrence was observed during 86 months of follow‐up.

Figure 1Mammographic and ultrasonographic findings. (a, b) Mammographic findings: (a) Case 1: A well‐defined mass is observed. (b) Case 4: A partially spherical, indistinct mass is noted. (c, d) Ultrasonographic findings: (c) Case 1: A complex solid and cystic mass is observed. (d) Case 2: A complex solid and cystic mass is observed.

**Figure:**
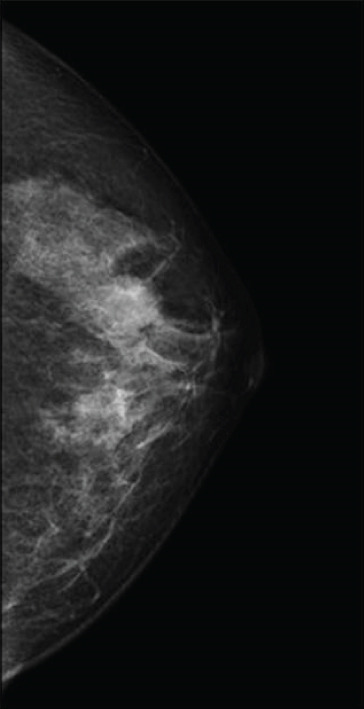
(a)

**Figure:**
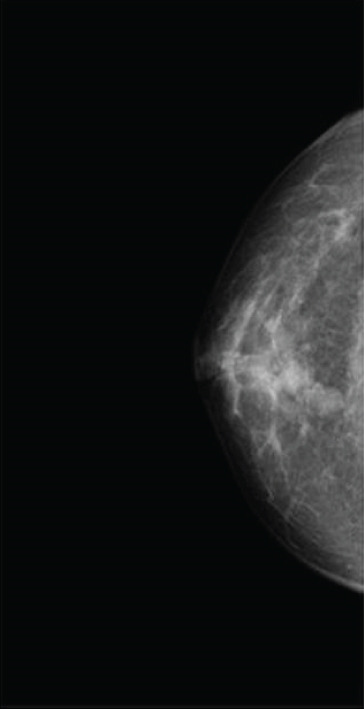
(b)

**Figure:**
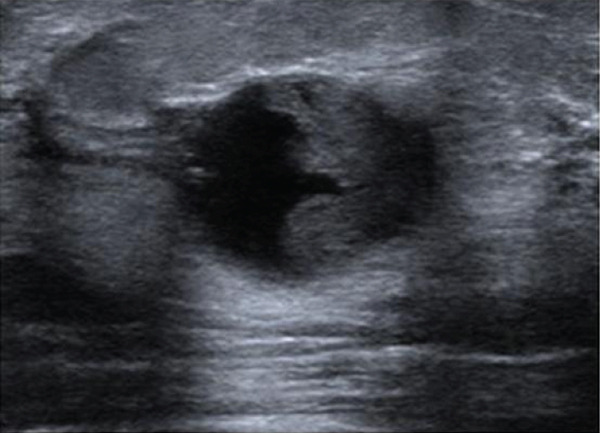
(c)

**Figure:**
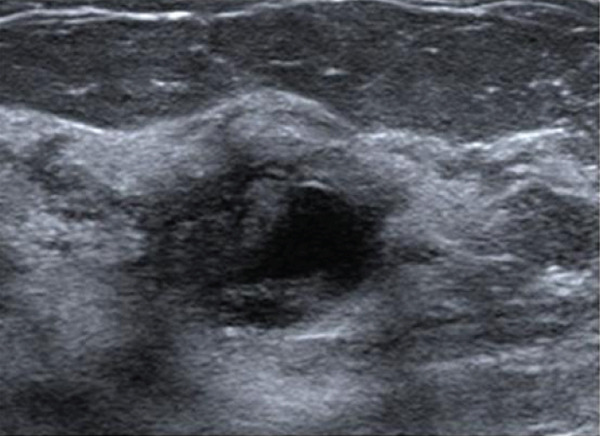
(d)

### 2.2. Case 2

A 38‐year‐old woman presented with a palpable subareolar mass in the left breast (12 o′clock position), measuring 15 mm. Mammography was unremarkable; however, ultrasonography revealed a complex mass with solid and cystic components (Figure 1d). CNB suggested a clear cell HA or low‐grade DCIS, and surgical excision was performed. The tumor displayed clear cells and cuboidal ductal cells with apocrine features. IHC was positive for p63 and CK7 and negative for ER. FISH demonstrated *MAML2* rearrangement. The patient remains recurrence‐free for 12 months.

### 2.3. Case 3

A 55‐year‐old woman was found to have an 8‐mm nonpalpable lesion in the left breast (12 o′clock position) during routine imaging. Mammography was negative; ultrasonography showed a hypoechoic mass with anechoic areas. Fine‐needle aspiration (FNA) suggested a malignant tumor. The tumor was excised and found to be composed of clear and eosinophilic cells arranged in solid–cystic patterns. IHC was positive for p63, CK5/6, and SOX10 (cuboidal cells) and negative for ER. *MAML2* rearrangement was confirmed by FISH. No recurrence has occurred over a 168‐month follow‐up period.

### 2.4. Case 4

A 43‐year‐old woman presented with abnormal nipple discharge and was found to have a 10‐mm retroareolar tumor in the right breast (3 o′clock position). Mammography showed an indistinct mass (Figure 1b), and ultrasonography revealed a cystic lesion with an intraductal appearance. FNA raised suspicion of malignancy. The excised specimen had two nodules, a main solid–cystic nodule with a small daughter nodule. An in situ component was not identified. Each nodule revealed a biphasic growth pattern with cuboidal ductal and clear cells. IHC showed positivity for CK5/6 and SOX10 (cuboidal cells) and negativity for ER. FISH revealed *MAML2* gene rearrangement. The patient remains disease‐free 170 months postoperatively.

All four tumors demonstrated solid and cystic architecture with apocrine features, which looked benign histologically. IHC profiles were consistent with adnexal origin, and *MAML2* gene rearrangement was confirmed in all cases by FISH. No evidence of malignant transformation or recurrence was observed during the long follow‐up period in any patient. Comparative histologic and immunohistochemical findings are detailed in Table 2.

## 3. Histopathological Findings

Macroscopically, all tumors were located within the mammary parenchyma and exhibited a well‐defined mass of firm white tissue (Figure 2a,b). Microscopically, all of them consisted of one or more well‐demarcated nodules that lacked connection with the epidermis and were surrounded by a thin fibrous capsule. No infiltrative growth into adjacent parenchyma was observed, supporting their benign nature. The tumors showed solid or solid–cystic proliferation with a papillary configuration (Figure 2c). The solid part was composed of two types of tumor cells with varying proportions. One type contained abundant, clear cytoplasm; small, round nuclei; inconspicuous nucleoli; and distinct cell membranes. The second component had fine granular, eosinophilic cytoplasm, round or oval nuclei, and indistinct cell membranes. The cystic lumens, surfaces of papillary projections, and duct‐like structures were lined by dark cuboidal cells with small nuclei and scant cytoplasm (Figures 2c, 2d, and 2e). A hyalinized stroma was also present in all cases (Figure 2d). Nuclear pleomorphism, necrosis, and mitotic figures were not observed. Periodic acid–Schiff (PAS) staining before and after diastase digestion confirmed glycogen in the clear cells.

Figure 2Gross and histopathologic features of breast hidradenoma. (a) Macroscopically, the tumor is a whitish, localized mass located within the mammary gland and composed of solid and cystic areas (Case 1). (b) This section shows one solid and cystic tumor and one small white daughter nodule in the mammary gland (Case 4). (c) Microscopic features showed a solid and cystic lesion. The tumor shows a two‐cell pattern, consisting of polygonal clear or acidophilic cells with distinct cell borders and dark cuboidal cells lining the lumen of cystic or glandular structures (Case 2). (d) Dark cuboidal cells lining the glandular structure and homogenous eosinophilic hyalinized stroma are noted (Case 4). (e) The daughter nodule in Case 4 shows histological features identical to the main nodule. (f) Immunophenotype of the lesional cells. The lesional cells are diffusely p63‐positive, but cuboidal cells lining the glandular structure are negative (Case 2). (g) SOX10 expression is limited to the low cuboidal cells lining the glandular structure (Case 1).

**Figure:**
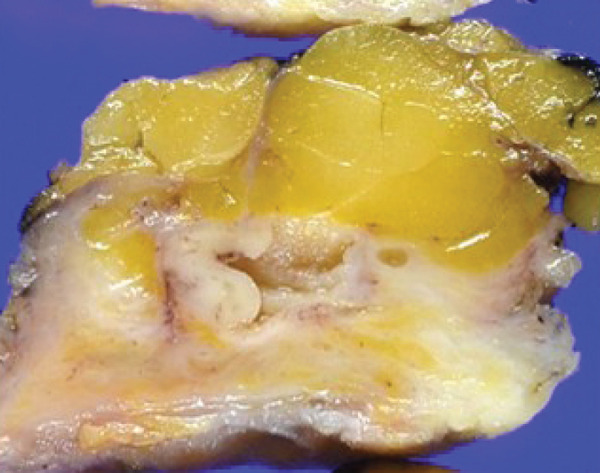
(a)

**Figure:**
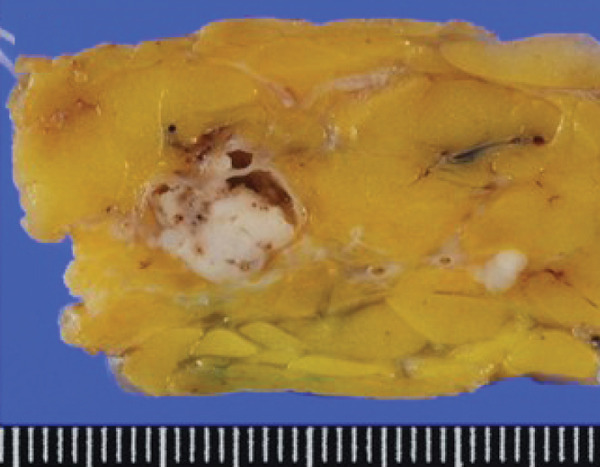
(b)

**Figure:**
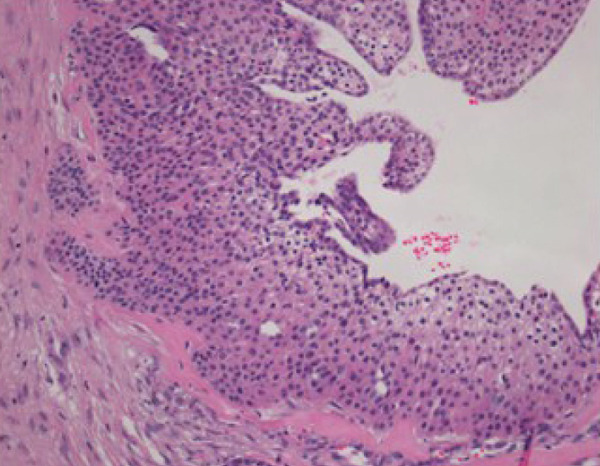
(c)

**Figure:**
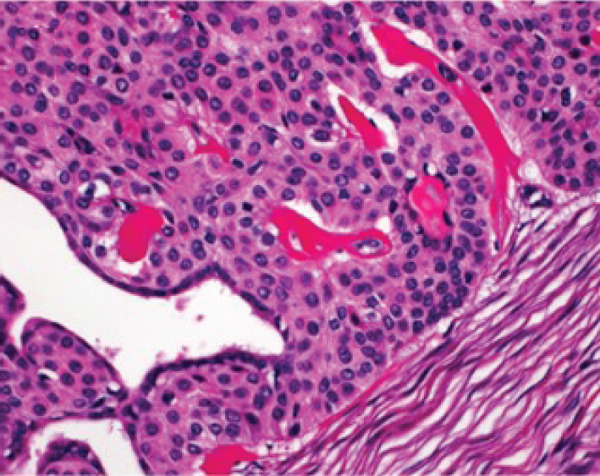
(d)

**Figure:**
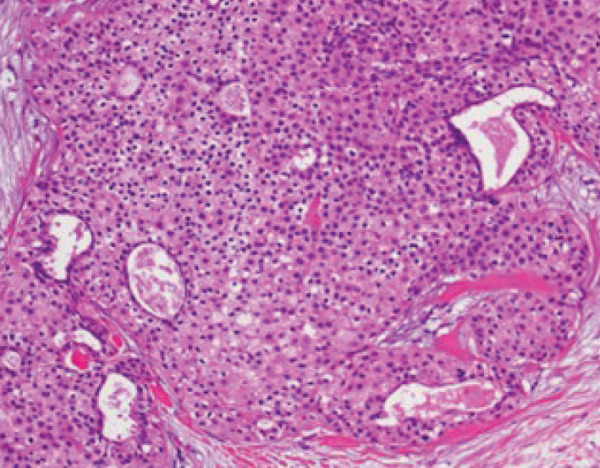
(e)

**Figure:**
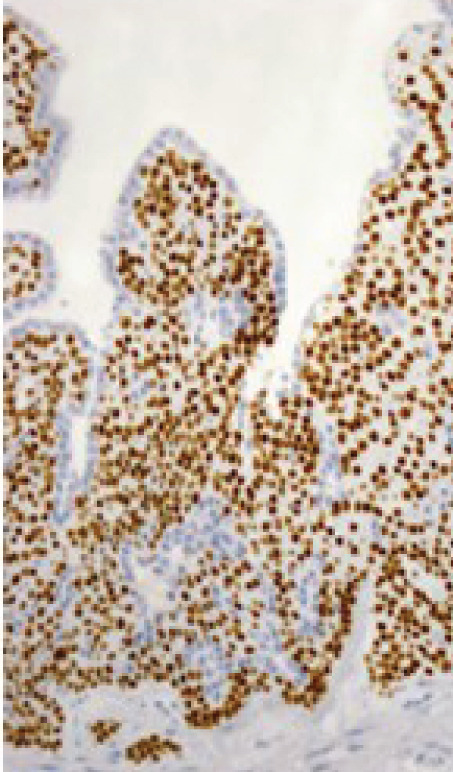
(f)

**Figure:**
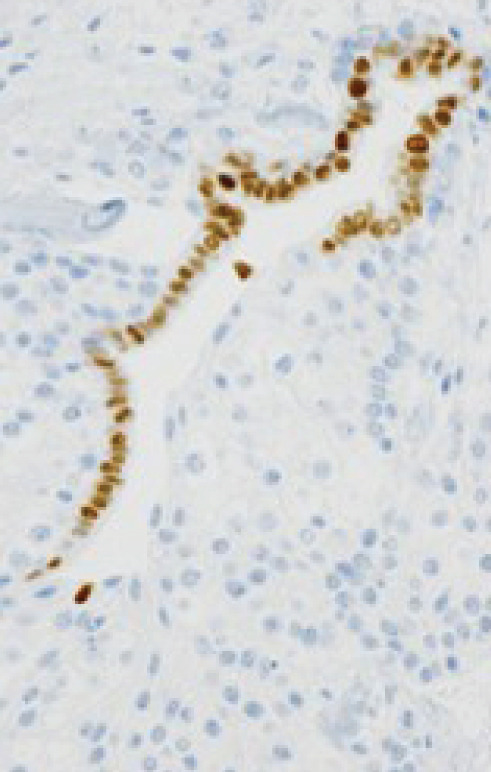
(g)

**Figure 3:**
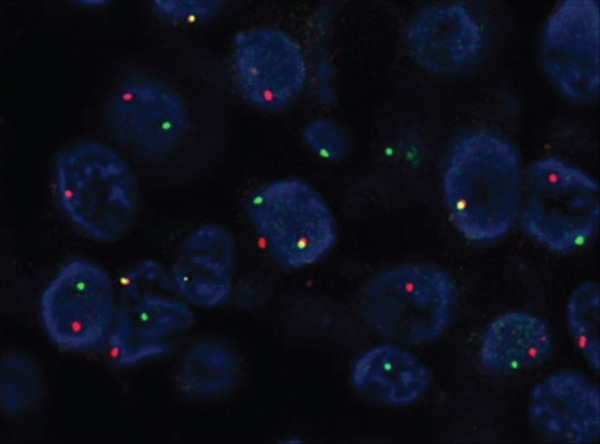
*MAML2* fluorescence in situ hybridization (FISH) analysis. Break‐apart FISH using dual‐color probes demonstrates separation of green (3 ^′^ *MAML2*) and red (5 ^′^ *MAML2*) signals in lesional cells, indicating the presence of a *MAML2* gene rearrangement. Case 1, original magnification ×1000.

Immunohistochemically, clear cells and eosinophilic cells were diffusely strongly positive for p63 (Figure 2f) and immunoreactive for CK7, AE1/3, CK5/6, and EMA, with some variability based on cell type. Myoepithelial markers such as calponin, SMA, and CD10 were negative. In contrast, the dark cuboidal cells lining the duct/glandular structures were negative for p63, and some luminal cells were SOX10‐positive (Figure 2g). These immunohistochemical findings highlight the complex adnexal differentiation of breast HA and further support its benign nature. Break‐apart FISH demonstrated *MAML2* gene rearrangement in all cases (Figure 3).

## 4. Discussion

HA is a relatively rare benign adnexal tumor that typically arises in the dermis, most often on the scalp, face, and extremities. In contrast, breast HA is extremely rare, with only 40 cases documented in the English literature to date [1–8]. At our institution, we observed four cases of intramammary HA, which represented 0.2% of benign breast lesions excised over a 21‐year period, underscoring its rarity in mammary tissue.

Clinically, HA typically presents as a painless breast mass, ranging in size from 0.7 to 7 cm. Other symptoms may also include nipple discharge, bluish discoloration of the overlying skin, or ulceration [15, 16]. Due to these overlapping clinical presentations, HA can often be misdiagnosed preoperatively as malignant. Consistent with previous reports, our patients presented with a painless mass and nipple discharge, reinforcing the importance of considering HA in the differential diagnosis of a breast mass.

On ultrasound, HA typically presents with both solid and cystic components, often appearing complex due to hemorrhage within the cystic area. In our study, three cases showed complex solid and cystic masses, and one case appeared as an intraductal lesion on ultrasonography, although it was located within the mammary parenchyma histologically.

This emphasizes that HA can mimic intraductal tumors on imaging, highlighting the variability and potential for misinterpretation in radiologic findings.

Even though pathological assessments have been performed among reported cases, a definitive diagnosis is rarely made by cytology or histology alone [17]. Previous studies have shown that only two cases were diagnosed preoperatively, with most diagnoses confirmed after resection [17, 18]. In our series, HA was suspected in two cases following CNB but was suspected as malignant in two cases examined by FNA, necessitating excisional biopsy for definitive diagnosis. It is very difficult to diagnose HA in advance, and sometimes it is misdiagnosed as malignant. Thus, some patients may be treated with unnecessary mastectomies or extensive local excisions. When imaging or histology could suggest HA as a differential, careful consideration should be given to avoid aggressive surgical interventions.

On microscopic examination, HA forms well‐circumscribed, lobulated masses subdivided by bands of sclerotic stroma. HA usually forms solitary masses and rarely multiple masses [19]. In our report, one case had multiple nodules, where accurate preoperative diagnosis might be more difficult to achieve.

Advances in molecular genetic techniques have provided greater insight into differentiating HA from other tumors. The fusion of *CRTC1* with *MAML2* has been reported in about 50% of HA of the skin and 50% of MEC of the salivary gland [9, 11]. In the breast, *MAML2* gene rearrangement has also been reported in MEC and HA, suggesting a shared molecular pathway in their tumorigenesis.

Because HA of the breast is a rare disease, only six genetic studies of breast HA have been reported in the English literature. An overview of the pathological findings of 13 cases in the previous papers and four cases in our report is shown in Table 2. All cases show solid and cystic lesions composed of clear and/or eosinophilic cells. Duct‐like cystic spaces and glandular structures lined by dark cuboidal cells are also characteristic. IHC, coexpression of CK5/6 and CK7, and strong p63 positivity are characteristic of HA. Immunoreactivity for p63 is an important diagnostic clue, serving as an initial step to exclude conventional breast carcinoma and to consider HA in the differential diagnosis. Furthermore, SOX10 was characterized as positive in p63‐negative cuboidal cells lining glandular structures. Homogenous eosinophilic material may also be a frequent finding.

The differential diagnosis of MEC and HA in the breast will become even more important in the future because of their similar histological and immunohistochemical profiles as well as their genetic backgrounds [20]. MEC is composed of mixed mucinous, intermediate, and squamous cells, in varying proportions. Solid and cystic patterns are also noted, as in HA, but cystic spaces are lined by mucous cells. The dark cuboidal cells were not evident in MEC cases. In contrast to HA, MECs show an invasive growth pattern. Although the immunophenotype of MEC and HA overlaps, a different staining pattern of SOX10 is useful. MEC shows diffuse SOX10 staining, but the expression is limited to the glandular cell population in HA. HA is a benign tumor, except for exceedingly rare cases, whereas MEC is an invasive tumor that recurs and metastasizes. Therefore, differentiation of these two based on pathological findings will be extremely important.

Our Case 4 was multinodular, which is unusual because most previously reported HAs were solitary. However, both nodules showed identical histological features of HA without any in situ component. Neither an infiltrative growth pattern nor mucin‐producing cells were observed. These findings support the diagnosis of HA rather than MEC.

The fusion gene between *CRTC1* and *MAML2* is attributed to t(11;19)(q21;p13) and has been identified in several cases of MEC of the salivary and bronchial glands [10, 21]. It acts as a transcription factor in the Notch and CREB regulatory pathways, inhibiting normal cell cycle and differentiation and contributing to tumorigenesis. This fusion gene was identified in cutaneous HA in 1994 [22], and 50% of skin was reported to have this fusion gene [9]. To date, 13 cases of HA of the breast with *MAML2* rearrangement have been reported [6–8, 12–14]. Consistent with findings by Black et al., where all breast HA cases were positive for *MAML2* break‐apart FISH [12], our study also found *MAML2* rearrangements in all four cases, suggesting a potential role of this fusion in breast HA. Additional cases are needed to validate these findings and clarify the significance of *MAML2* in breast HA. However, it should be noted that the presence of *MAML2* rearrangements is not useful for distinguishing between HA and MEC.

In summary, this study highlights the clinical and histologic characteristics of breast HA and the frequent presence of *MAML2* rearrangements, which may contribute to accurate diagnosis and potentially avoid overtreatment. Our findings suggest that breast HA, while rare, should be considered in the differential diagnosis of complex breast masses to prevent misinterpretation as malignant lesions.

## Ethics Statement

This study was conducted in accordance with the Declaration of Helsinki and was approved by the Institutional Review Board of Sagara Hospital (approval no. 25‐2). Informed consent was waived as no identifiable personal information was included.

## Disclosure

All authors read and approved the final manuscript.

## Conflicts of Interest

The authors declare no conflicts of interest.

## Author Contributions

N.G. and Y.O. conceived and designed the study. N.G. collected clinical data. Y.O., T.H., and A.I.H. performed pathological review and analysis. N.G. and Y.O. drafted the manuscript. S.K., Y.S., and S.O. critically revised the manuscript for intellectual content.

## Funding

No funding was received for this manuscript.

## Data Availability

The data that support the findings of this study are available from the corresponding author upon reasonable request.
